# The Editor's Letter-Box

**Published:** 1921-04-30

**Authors:** 


*To the Editor of* The Hospital.

Sir,—The letter which you publish from " One of a Large Family " suggests that the writer would be glad to learn the opinions of some others among your readers. Judging by the public literature now available, some of which you have on occasion criticised—almost too mildly perhaps—your correspondent has, in any case, ample opportunity of learning what others think upon this all-important matter.

Few will disagree that a moderate practice of birth-control is necessary. As you say, the required knowledge is almost universally prevalent. The size of the family should, from both economic and hygienic standards, be graded to the power of the parents to support it. If the national average approximated to this requirement there would be little need to talk of race-suicide. The present birth-rate hardly suggests that there is. Nevertheless, it is obvious that a family can be too large, and its members, individually and indirectly, suffer in consequence. But surely that is no reason to fly from one extreme to the other, which an easily influenced public is only too liable to do in the face of propaganda such as fashion now tolerates. Provident deliberation is a perfectly necessary proceeding, provided that parents are willing to rear such families as their means allow. The large family is only a misfortune if family-purse signally fails to keep pace with it.—Yours, etc.,

A General Practitioner.

